# Supplementary material for: Association between Biomarkers (VEGF-R2, VEGF-R3, VCAM-1) and Treatment Duration in Patients with Neuroendocrine Tumors Receiving Therapy with First-Generation Somatostatin Analogues
Source: Biomedicines. 2023 Mar 10;11(3):842. doi: 10.3390/biomedicines11030842 (PMC10044914; doi:10.3390/biomedicines11030842)
Supplement: Supplementary file 1 [file biomedicines-11-00842-s001.zip › Supplementary Material corrected 8.03.2023.pdf]

Supplementary Material

Association between Biomarkers (VEGF-R2, VEGF-R3, VCAM-1), and Treatment Duration in Neuroendocrine Tumors Patients Receiving Therapy with First-Generation Somatostatin Analogues

Violetta Rosiek *, Ksenia Janas and Beata Kos-Kudła

Department of Endocrinology and Neuroendocrine Tumors, Department of Pathophysiology and Endocrinology, Medical University of Silesia, 40-514 Katowice, Poland; Ksenia.janas@sum.edu.pl (K.J.); bkoskudla@sum.edu.pl (B.K.-K.)

* Correspondence: vrosiek@sum.edu.pl

1. Supplemental Tables

Table S1. Vascular endothelial growth factor receptor 2 (VEGF-R2), vascular endothelial growth factor receptor 3 (VEGF-R3), and vascular cell adhesion molecule-1 (VCAM-1) in patients with neuroendocrine tumors (NET) treated with somatostatin analogues (SSAs).

**Variable**	**Group B n = 56**	**Group C n = 55**	**Group D n = 46**	**Group E n = 35**	**Group F n = 26**	**Group G n = 22**	**Friedman test (chi-square) test**	
				Median (IR)			$\chi 2$	p
**VEGF-R2 (pg/mL)**	7717 (6567-8662)	7150 (6420-8109)	7067 (6430-8244)	6541 (5747-8193)	6113 (5157-8751)	6345 (5781-7751)	84.60	< 0.001
**VEGF-R3 (ng/mL)**	65 (59-73)	65 (46-81)	67 (58-80)	66 (48-75)	60 (51-66)	54 (46-66)	83.66	< 0.001
**VCAM-1 (ng/mL)**	667 (595-838)	764 (605-978)	803 (630-946)	688 (587-1036)	675 (565-785)	693 (554-816)	88.86	< 0.001

* Results are presented as median (and quartiles), where appropriate. Abbreviations: see table 2.

Table S2. Spearman Rank Correlations. Spearman's coefficients of the relationships among analyzed variables in NET patients: (red correlations statistically significant, p<0.05).

Variables	Treatment duration (months)	VEGF-R2 (pg/mL)	VEGF-R3 (ng/mL)	VCAM-1 (ng/mL)
Treatment duration (months)	1.00	**-0.19**	-0.07	-0.05
VEGF-R2 (pg/mL)	-0.19	1.00	**0.42**	**-0.26**
VEGF-R3 (ng/mL)	-0.07	0.42	1.00	**0.29**
VCAM-1 (ng/mL)	-0.05	-0.26	0.29	1.00

Abbreviations: VEGF-R2, vascular endothelial growth factor receptor 2; VEGF-R3, vascular endothelial growth factor receptor 3; VCAM-1, vascular cell adhesion molecule 1.

Table S3. The capacity of vascular endothelial growth factor receptor 2 (VEGF-R2), vascular endothelial growth factor receptor 3 (VEGF-R3), and vascular cell adhesion molecule-1 (VCAM-1) for differentiating SSA-treated vs. non-treated NET patients.

Variable	AUC	SE	95% CI	p
**VEGF-R2** (pg/mL)				
Group B vs. Group C	0.42	0.05	0.31-0.52	NS
Group B vs. Group D	0.43	0.06	0.31-0.54	NS
Group B vs. Group E	0.37	0.06	0.25-0.50	**0.046**
Group B vs. Group F	0.36	0.08	0.21-0.50	NS
Group B vs. Group G	0.30	0.07	0.17-0.43	**0.003**
**VEGF-R3** (ng/mL)				
Group B vs. Group C	0.51	0.06	0.40-0.62	NS
Group B vs. Group D	0.55	0.06	0.44-0.66	NS
Group B vs. Group E	0.50	0.06	0.38-0.63	NS
Group B vs. Group F	0.42	0.07	0.29-0.56	NS
Group B vs. Group G	0.37	0.08	0.21-0.53	NS
**VCAM-1** (ng/mL)				
Group B vs. Group C	0.60	0.05	0.49-0.71	NS
Group B vs. Group D	0.61	0.06	0.50-0.72	**0.048**
Group B vs. Group E	0.55	0.07	0.42-0.68	NS
Group B vs. Group F	0.45	0.07	0.31-0.59	NS
Group B vs. Group G	0.50	0.07	0.36-0.64	NS

Abbreviations: NS, not significant, CI, confidential interval, AUC, area under the curve; SE, standard error.

Table S4. Vascular endothelial growth factor receptor 2 (VEGF-R2), vascular endothelial growth factor receptor 3 (VEGF-R3), and vascular cell adhesion molecule-1 (VCAM-1) levels depending on the grade, and stage of the disease.

Variable		Median	IR	p-value*
**VEGF-R2** (pg/mL)				
Tumor grade	G1	7799	6766 - 8636	NS
	G2	7297	6006 - 8845	
Tumor grade, final measurement	G1	6346	5588 - 7814	NS
	G2	6343	6024 - 6627	
Stage of disease	SD	7824	6653 - 8587	NS
	PD	7712	6463 – 8845	
Stage of disease, final measurement	SD	5925	4809 - 7304	NS
	PD	6346	5781 - 7814	

**VEGF-R3** (ng/mL)				
Tumor grade	G1	67	60 - 73	NS
	G2	60	47 – 73	
Tumor grade, final measurement	G1	53	46 – 83	NS
	G2	55	51 - 60	
Stage of disease	SD	62	47 – 72	NS
	PD	68	60 – 75	
Stage of disease, final measurement	SD	53	46 – 66	NS
	PD	55	46 - 83	
**VCAM-1** (ng/mL)				
Tumor grade	G1	664	582 – 835	NS
	G2	680	608 - 842	
Tumor grade, final measurement	G1	678	544 - 765	NS
	G2	816	675 - 1143	
Stage of disease	SD	700	506 - 773	NS
	PD	664	609 – 872	
Stage of disease, final measurement	SD	846	554 - 1015	NS
	PD	678	544 - 765	

* Mann–Whitney test; Abbreviations: IR, interquartile range; PD, progressive disease; SD, stable disease.

2. Supplemental Figures

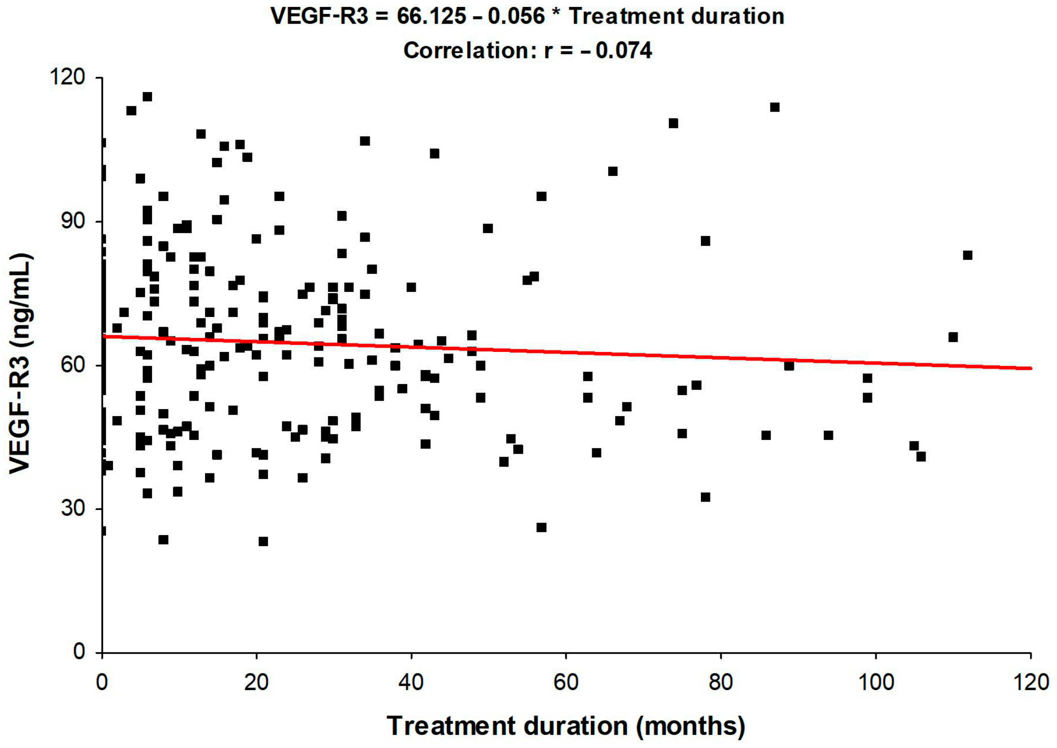

Figure S1. Spearman's Rank Correlations. Spearman's coefficients of the relationships among analyzed variables in NET patients: VEGF-R3 vs. Treatment duration (the presented correlation is not statistically significant, p>0.05).

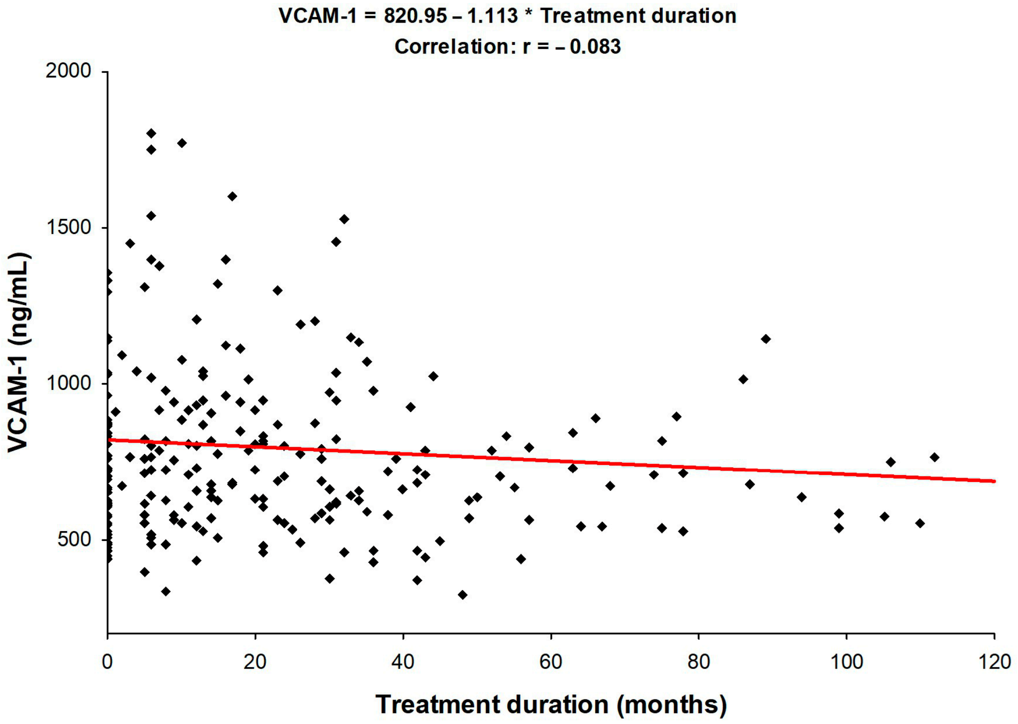

Figure S2. Spearman's Rank Correlations Spearman's coefficients of the relationships among analyzed variables in NET patients: VCAM-1 vs. Treatment duration (the presented correlation is not statistically significant, p> 0.05).
