# Supplementary figures and images for: Association between Biomarkers (VEGF-R2, VEGF-R3, VCAM-1) and Treatment Duration in Patients with Neuroendocrine Tumors Receiving Therapy with First-Generation Somatostatin Analogues
Source: Biomedicines. 2023 Mar 10;11(3):842. doi: 10.3390/biomedicines11030842 (PMC10044914; doi:10.3390/biomedicines11030842)

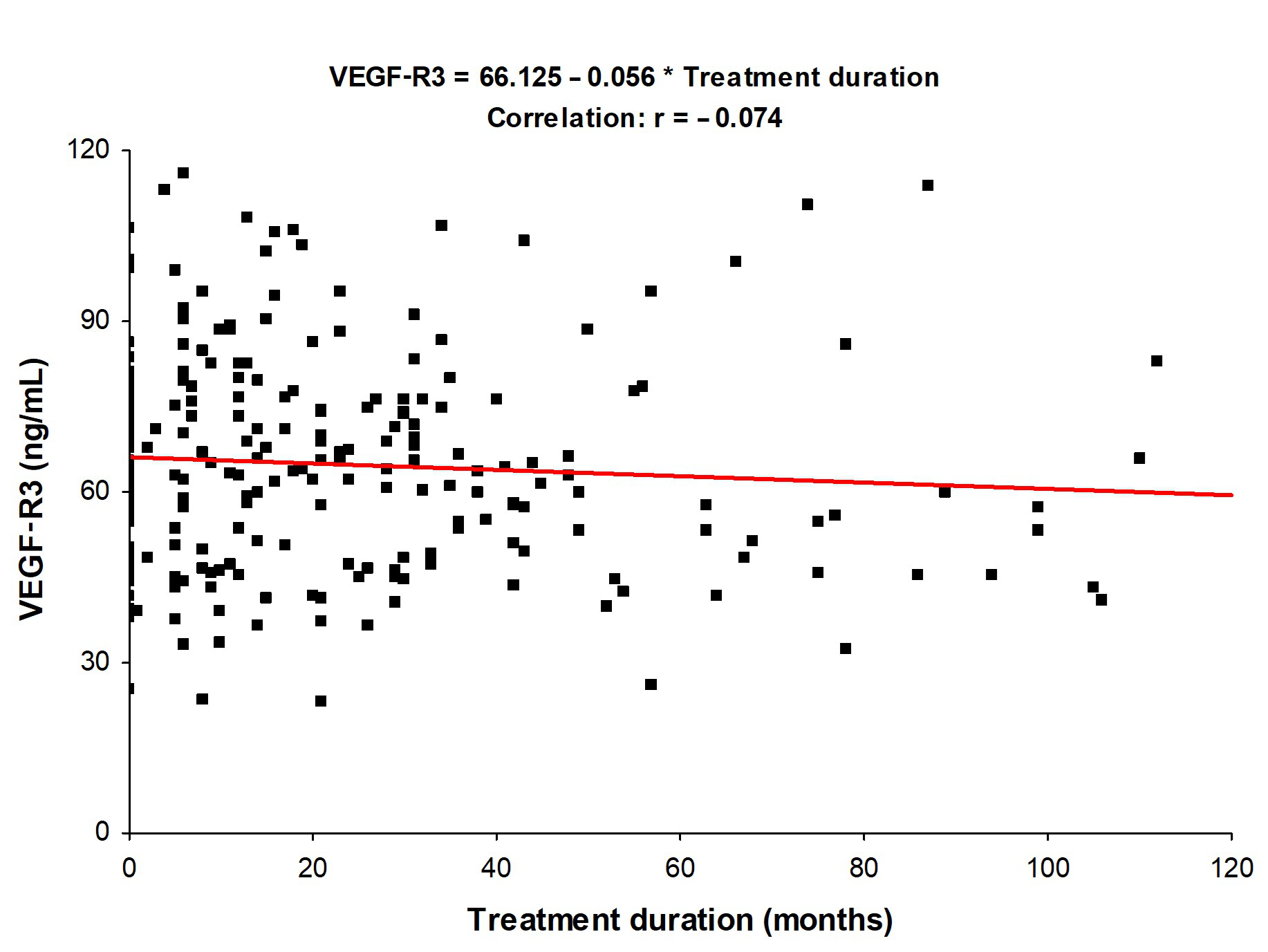

Supplement: Supplementary file 1 [file biomedicines-11-00842-s001.zip › Figure S1.jpg]

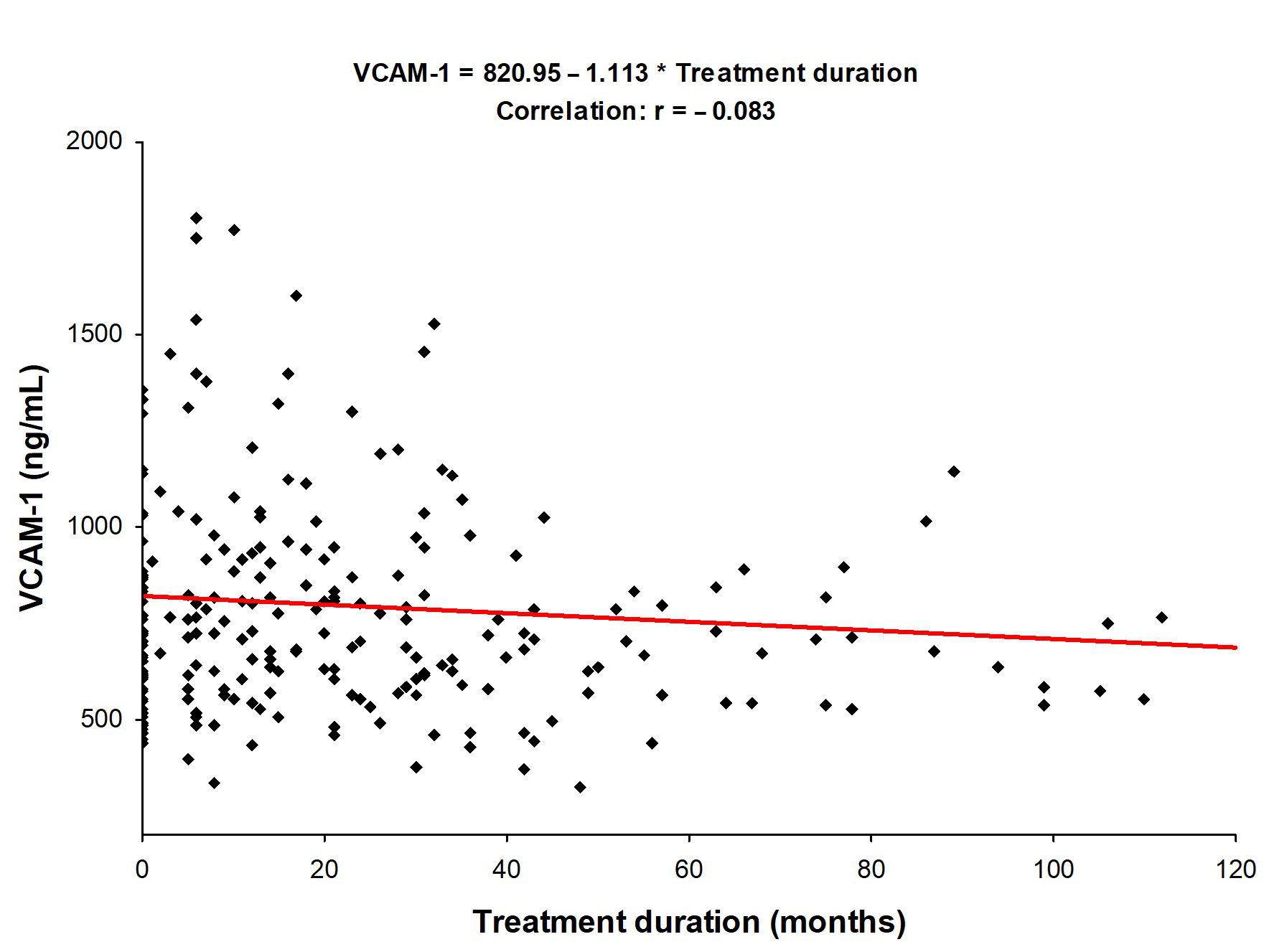

Supplement: Supplementary file 1 [file biomedicines-11-00842-s001.zip › Figure S2.jpg]
